# Comparison of Radiation Exposure Among Interventional Echocardiographers, Interventional Cardiologists, and Sonographers During Percutaneous Structural Heart Interventions

**DOI:** 10.1001/jamanetworkopen.2022.20597

**Published:** 2022-07-07

**Authors:** David A. McNamara, Rajus Chopra, Jeffrey M. Decker, Michael W. McNamara, Stacie M. VanOosterhout, Duane C. Berkompas, Musa I. Dahu, Mohamad A. Kenaan, Wassim I. Jawad, William M. Merhi, Jessica L. Parker, Ryan D. Madder

**Affiliations:** 1 Frederik Meijer Heart & Vascular Institute, Spectrum Health, Grand Rapids, Michigan

## Abstract

**Importance:**

Transesophageal echocardiography during percutaneous left atrial appendage closure (LAAO) and transcatheter edge-to-edge mitral valve repair (TEER) require an interventional echocardiographer to stand near the radiation source and patient, the primary source of scatter radiation. Despite previous work demonstrating high radiation exposure for interventional cardiologists performing percutaneous coronary and structural heart interventions, similar data for interventional echocardiographers are lacking.

**Objective:**

To assess whether interventional echocardiographers are exposed to greater radiation doses than interventional cardiologists and sonographers during structural heart procedures.

**Design, Setting, and Participants:**

In this single-center cross-sectional study, radiation doses were collected from interventional echocardiographers, interventional cardiologists, and sonographers at a quaternary care center during 30 sequential LAAO and 30 sequential TEER procedures from July 1, 2016, to January 31, 2018. Participants and study personnel were blinded to radiation doses through data analysis (January 1, 2020, to October 12, 2021).

**Exposures:**

Occupation defined as interventional echocardiographers, interventional cardiologists, and sonographers.

**Main Outcomes and Measures:**

Measured personal dose equivalents per case were recorded using real-time radiation dosimeters.

**Results:**

A total of 60 (30 TEER and 30 LAAO) procedures were performed in 60 patients (mean [SD] age, 79 [8] years; 32 [53.3%] male) with a high cardiovascular risk factor burden. The median radiation dose per case was higher for interventional echocardiographers (10.6 μSv; IQR, 4.2-22.4 μSv) than for interventional cardiologists (2.1 μSv; IQR, 0.2-8.3 μSv; *P* < .001). During TEER, interventional echocardiographers received a median radiation dose of 10.5 μSv (IQR, 3.1-20.5 μSv), which was higher than the median radiation dose received by interventional cardiologists (0.9 μSv; IQR, 0.1-12.2 μSv; *P* < .001). During LAAO procedures, the median radiation dose was 10.6 μSv (IQR, 5.8-24.1 μSv) among interventional echocardiographers and 3.5 (IQR, 1.3-6.3 μSv) among interventional cardiologists (*P* < .001). Compared with interventional echocardiographers, sonographers exhibited low median radiation doses during both LAAO (0.2 μSv; IQR, 0.0-1.6 μSv; *P* < .001) and TEER (0.0 μSv; IQR, 0.0-0.1 μSv; *P* < .001).

**Conclusions and Relevance:**

In this cross-sectional study, interventional echocardiographers were exposed to higher radiation doses than interventional cardiologists during LAAO and TEER procedures, whereas sonographers demonstrated comparatively lower radiation doses. Higher radiation doses indicate a previously underappreciated occupational risk faced by interventional echocardiographers, which has implications for the rapidly expanding structural heart team.

## Key Points

**Question:**

Are interventional echocardiographers exposed to greater occupational radiation doses than interventional cardiologists and sonographers during structural heart procedures?

**Findings:**

In this cross-sectional study of 60 structural heart procedures, interventional echocardiographers experienced higher head-level radiation doses than interventional cardiologists and sonographers.

**Meaning:**

These comparatively higher radiation doses indicate a previously underappreciated occupational risk faced by interventional echocardiographers, which has implications for the rapidly expanding structural heart team.

## Teaser

This cross-sectional study compares head-level radiation doses for interventional echocardiographers, interventional cardiologists, and sonographers during left atrial appendage closure and transcatheter edge-to-edge mitral valve repair procedures.

## Introduction

During the past decade, percutaneous procedures to treat structural heart disease have rapidly expanded. Among structural heart interventions, left atrial appendage occlusion (LAAO) and transcatheter edge-to-edge mitral valve repair (TEER) are now performed routinely at many centers and are increasing rapidly.^1,2^ Both LAAO and TEER are typically performed under the guidance of fluoroscopy and transesophageal echocardiography (TEE), which has resulted in the addition of an interventional echocardiographer as an integral member of the structural heart team.

Because there is a need for intermittent manipulation of the TEE probe during LAAO and TEER, the interventional echocardiographer must stand near the patient, which is the principal source of scatter radiation during fluoroscopically guided procedures. Long-term exposure to scatter radiation in the cardiac catheterization laboratory has been associated with multiple adverse health effects among interventional cardiologists, including premature cataract formation,^3,4^ early carotid atherosclerosis,^5^ and possibly left-sided brain malignant tumors.^6^ Whether interventional echocardiographers on the structural heart team are exposed to levels of radiation sufficient to pose similar adverse health risks as those faced by interventional cardiologists is not yet known. Given the rapidly increasing rates of LAAO^7^ and TEER^8^ procedures and progress in the field of interventional echocardiography, steps to quantify the occupational risk of interventional echocardiographers are warranted to help inform mitigation attempts.

This study aims to compare head-level radiation doses for interventional echocardiographers with those of interventional cardiologists and sonographer controls during LAAO and TEER procedures. Given the nearness of interventional echocardiographers to the radiation emission source, we hypothesized that interventional echocardiographers would be exposed to higher radiation doses compared with interventional cardiologists and sonographers.

## Methods

### Study Population

This single-center, prospective, investigator-initiated cross-sectional study was designed to investigate radiation doses to interventional echocardiographers during LAAO and TEER procedures. The study was conceived, designed, and conducted by investigators of the Frederik Meijer Heart & Vascular Institute of Spectrum Health (Grand Rapids, Michigan) as previously described.^9^ Interventional cardiologists and interventional echocardiographers were board-certified cardiologists with a mean of 10 and 3 years, respectively, of clinical practice beyond completion of their fellowship training; trainees were not involved during cases. Data on race and ethnicity were not collected as a part of this study. The institutional review board at Spectrum Health approved the protocol, and all participants provided verbal informed consent. This study followed the Strengthening the Reporting of Observational Studies in Epidemiology (STROBE) reporting guideline.

**Figure 1.**
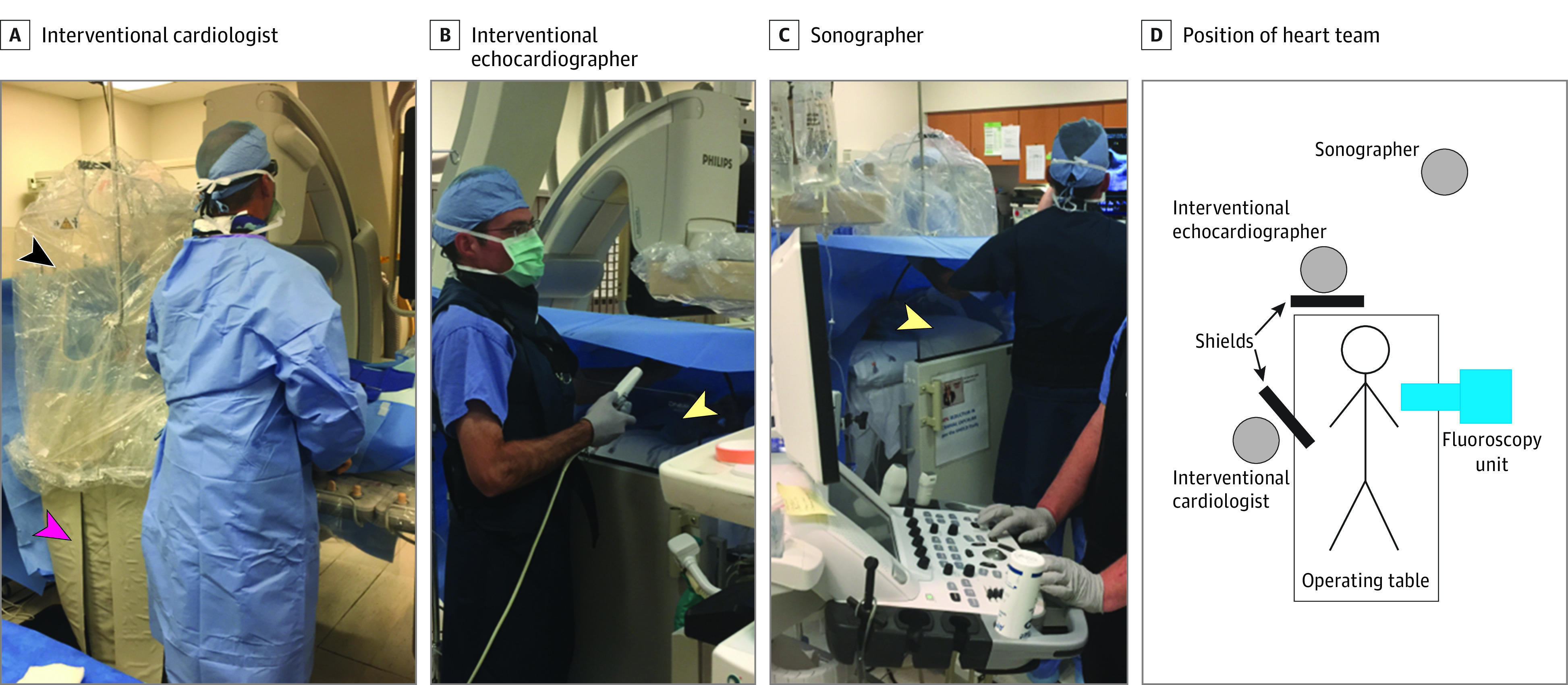
Interventional Echocardiographer, Interventional Cardiologist, and Sonographer During Fluoroscopically Guided Structural Heart Interventions Left atrial appendage closure and transcatheter edge-to-edge mitral valve repair were performed with an interventional cardiologist standing immediately adjacent to the procedure table. A, Interventional cardiologists used a ceiling-mounted upper-body lead shield (black arrow) and a lower-body lead shield attached to the side of the operating table extending from the table to the floor (red arrow). B, The interventional echocardiographer stood at the patient’s head to manipulate the transesophageal echocardiogram probe during the procedure. Interventional echocardiographers used a mobile, height-adjustable, accessory lead shield (yellow arrow). The upper section of the shield was raised to a height that allowed the interventional echocardiographer to extend their arms over the shield to manipulate the transesophageal echocardiogram probe throughout the case. C, A sonographer (right), who assisted with image acquisition throughout the procedure, stood approximately 250 cm from the radiation source. The nearness of the interventional echocardiographer to the radiation source is evident in this image. D, Overhead diagram shows the relative position of the interventional cardiologist, interventional echocardiographer, and sonographer to the patient.

Radiation exposure data were prospectively collected on 30 consecutive LAAO procedures and 30 consecutive TEER procedures performed between July 1, 2016, and January 31, 2018. Details of implantation protocols for these commercially available devices have been previously published.^9,10^ State-of-the art fluoroscopy systems (AlluraClarity, Philips) with real-time image noise reduction technology (Clarity IQ, Philips) were used. In all cases, fluoroscopy was performed at a frame rate of 15 frames per second. Fluoroscopy and cineangiography were used according to operator discretion. All LAAO procedures involved implantation of a commercially available closure device (WATCHMAN, Boston Scientific). All TEER procedures were performed using a commercially available percutaneous edge-to-edge repair device (MitraClip, Abbott Vascular). Procedures were performed using a transseptal approach and were guided by fluoroscopy and TEE. For the TEER and LAAO procedures, the measured direct-line distances from the radiation source to team member while in a neutral position were 26 cm for the interventional echocardiographer, 36 cm for the interventional cardiologist, and 250 cm for the sonographer. The procedure rooms were arranged as shown in Figure 1.

### Radiation Monitoring

Real-time radiation exposure data were collected using a commercially available dosimetry system that contains a bedside monitor capable of displaying real-time radiation doses (RaySafe i2, Unfors RaySafe). To measure head-level radiation doses, each physician and staff member wore a dosimeter located on the left anterior side of the glasses or on the left anterior side of the thyroid collar as previously described.^11,12^ This system collects the cumulative head-level radiation exposure per case. Physicians and sonographers were blinded to the radiation data collected by the dosimeters for the duration of the study.

### Radiation Protection

Interventional echocardiographers, interventional cardiologists, and sonographers wore traditional lead apparel, consisting of a lead skirt, apron, and thyroid collar. A mobile, height-adjustable, accessory lead shield was positioned between the patient and interventional echocardiographer. The lead shield used in this study included a fixed lower half with a height-adjustable upper half of the shield, which provided increased shielding while the transesophageal echocardiography probe was not being manipulated but could be lowered during probe manipulation (Figure 1; see eAppendix in the Supplement for details). According to standard operating procedure at the study institution, 2 shields were positioned between the patient and interventional cardiologist in all cases: a ceiling-mounted upper-body lead shield with a patient contour cutout and a lower-body lead shield attached to the side of the operating table extending from table to floor (Figure 1). The sonographer did not have a separate, dedicated mobile lead shield.

### Radiation Doses

The primary measure of interest in this study was the personal dose equivalent (H_p_[10]) as recorded by dosimeters worn by interventional echocardiographers, interventional cardiologists, and sonographers and reported directly by the dosimetry system. In addition to reporting the personal dose equivalent per case, the frequency of a personal dose equivalent greater than 20 μSv was also reported because this dose is roughly 10-fold higher than the mean personal dose equivalent received by interventional cardiologists across a variety of procedures.^13^ Additional radiation metrics recorded for each case included the fluoroscopy time, air kerma (AK), and dose area product (DAP), which were automatically calculated by the fluoroscopy imaging system. Both AK and DAP are commonly used metrics to estimate patient radiation dose; AK is defined as the radiation delivered to air at a reference point located 15 cm on the x-ray tube side of isocenter, and DAP is defined as the product of AK and the x-ray field area.^14^

### Statistical Analysis

Data analyses were conducted between January 1, 2020, and October 12, 2021. Descriptive statistics were used to summarize baseline characteristics and outcome measures. Normally distributed continuous variables are given as mean (SD). Nonnormally distributed continuous variables are given as median (IQR). Categorical variables are given as number (percentage). Normality was assessed by visual inspection of histogram plots, the Shapiro-Wilk *P* value, and ensuring the skewness and kurtosis values fell between −2 and 2. *P* values for comparison of continuous variables across the 3 occupations were derived from pairwise comparisons using the Wilcoxon rank sum test with a Bonferroni correction used to adjust the α for those tests as appropriate. *P* values for comparison of continuous variables between the 2 procedure types (LAAO and TEER) were derived from 2-sample independent *t* tests if data were normally distributed or from Wilcoxon rank sum tests if data were not normally distributed. *P* values for comparison of categorical variables were generated with a χ^2^ analysis or a Fisher exact test if the expected cell counts were below 5 in more than 20% of the cells. Because there were 3 different groups, a Bonferroni correction was applied to these tests if needed when assessing the data across the 3 occupations. A 2-sided *P* < .05 was considered to be statistically significant unless otherwise noted. Odds ratios for the categorical comparisons were produced to assess the association for those that were significant. No data were imputed if missing. All statistical analyses were generated using SAS software, version 7.1 (SAS Institute Inc), and the statistical figures were generated using R statistical software, version 4.0.2 (R Foundation for Statistical Computing).

## Results

### Study Population

A total of 60 (30 TEER and 30 LAAO) procedures were performed in 60 patients (mean [SD] age, 79 [8] years; 28 [46.7%] female and 32 [53.3%] male; mean [SD] body mass index [calculated as weight in kilograms divided by height in meters squared], 29.4 [6.1]) with a high cardiovascular risk factor burden. Additional baseline characteristics of the patients are noted in Table 1. Procedural radiation metrics are presented in Table 2. Despite higher fluoroscopic time during TEER cases, no differences in radiation doses (AK or DAP) were observed between the LAAO and TEER procedures.

**Table 1.** Baseline Characteristics of Patients^a^

Characteristic	Overall (N = 60)	LAAO (n = 30)	TEER (n = 30)	*P* value^b^
Age, mean (SD), y	79 (8)	80 (6)	78 (9)	.39
Sex				
Male	32 (53.3)	17 (56.7)	15 (50.0)	.60
Female	28 (46.7)	13 (43.3)	15 (50.0)	
Height, mean (SD), cm	167.9 (12.3)	168.2 (12.0)	167.7 (12.8)	.89
Weight, mean (SD), kg	83.5 (21.9)	87.7 (23.3)	79.3 (19.9)	.14
BMI, mean (SD)	29.4 (6.1)	30.7 (5.8)	28.1 (6.1)	.10
BSA, mean (SD), m^2^	1.96 (0.30)	2.01 (0.32)	1.91 (0.28)	.23
Hypertension	46 (76.7)	23 (76.7)	23 (76.7)	.99
Diabetes	13 (21.7)	5 (16.7)	8 (26.7)	.35
Coronary artery disease	36 (60.0)	17 (56.7)	19 (63.3)	.60
Cerebrovascular accident	13 (21.7)	10 (33.3)	3 (10.0)	.03

Abbreviations: BMI, body mass index (calculated as weight in kilograms divided by height in meters squared); BSA, body surface area; LAAO, percutaneous left atrial appendage closure; TEER, transcatheter edge-to-edge mitral valve repair.

^a^ Data are presented as number (percentage) of patients unless otherwise indicated.

^b^ *P* values compare the LAAO and TEER groups.

**Table 2.** Radiation Metrics During Percutaneous LAAO and TEER

Metric	Median (IQR)			*P* value^a^
	All (N = 60)	LAAO (n = 30)	TEER (n = 30)	
Fluoroscopy time, min	13.4 (9.1-21.2)	9.2 (6.9-14.2)	20.9 (12.6-26.0)	<.001
Air kerma, mGy	136 (80-260)	164 (97-268)	109 (51-164)	.07
DAP, mGy × cm^2^	20.2 (11.3-30.9)	22.6 (12.9-28.8)	17.6 (7.9-33.7)	.54

Abbreviations: DAP, dose area product; LAAO, percutaneous left atrial appendage closure; TEER, transcatheter edge-to-edge mitral valve repair.

^a^ *P* values compare the LAAO and TEER groups.

### Interventional Echocardiographer Radiation Doses

**Figure 2.**
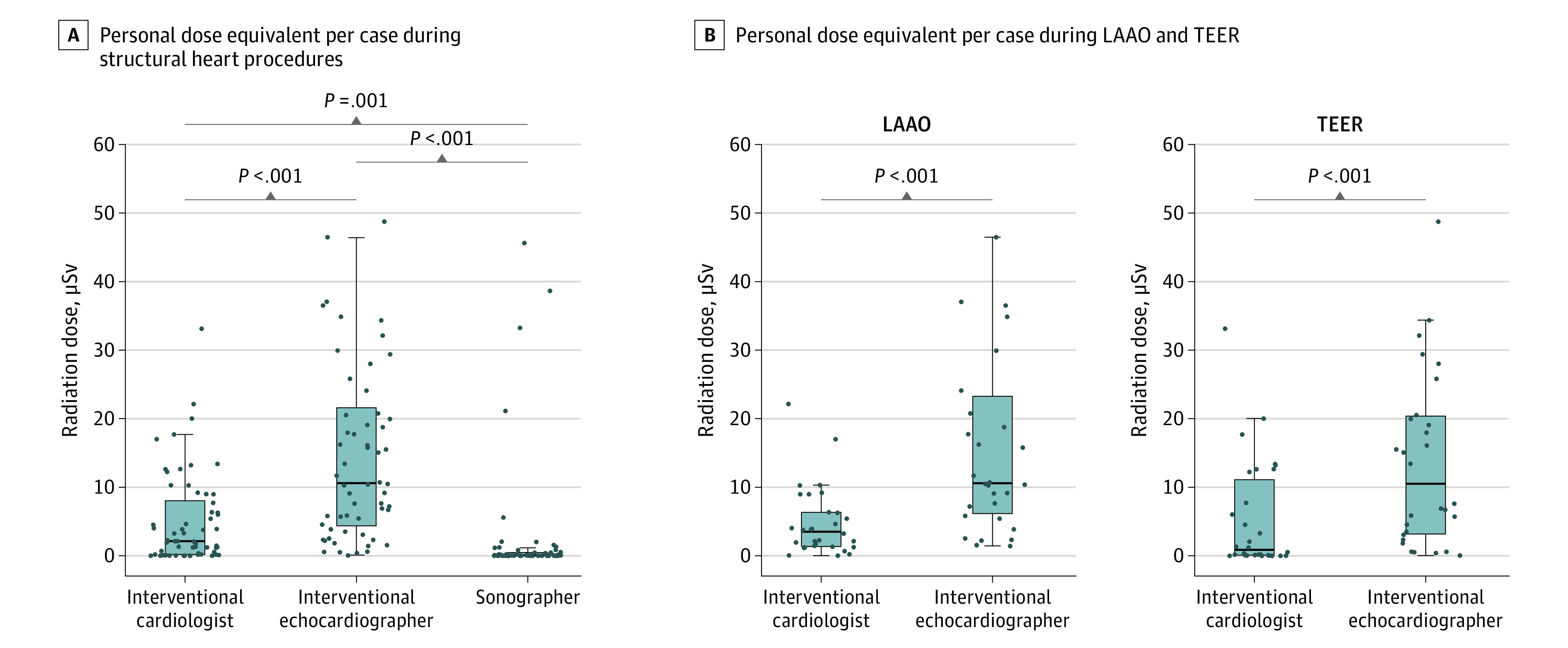
Radiation Dose for Interventional Echocardiographers, Interventional Cardiologists, and Sonographers During Fluoroscopically Guided Structural Heart Interventions A, Distribution of personal dose equivalent per case for interventional cardiologists, interventional echocardiographers, and sonographers during structural heart procedures (n = 60). B, Distribution of personal dose equivalent per case for the interventional cardiologist and interventional echocardiographer during percutaneous left atrial appendage occlusion (LAAO) (n = 30) and percutaneous transcatheter edge-to-edge mitral valve repair (TEER) (n = 30) are shown.

Among the 60 structural heart interventions, the median per case radiation dose was 10.6 μSv (IQR, 4.2-22.4 μSv) for interventional echocardiographers and 2.1 μSv (IQR, 0.2-8.3 μSv) for interventional cardiologists (*P* < .001) (Figure 2). The odds of interventional echocardiographers having a radiation dose greater than 20 μSv were 7.5 (95% CI, 2.1-27.3) times greater than interventional cardiologists (*P* < .001). No differences in median radiation doses between TEER and LAAO procedures were present for interventional echocardiographers (10.5 μSv [IQR, 3.1-20.5 μSv] vs 10.6 μSv [IQR, 5.8-24.1 μSv]; *P* = .36), interventional cardiologists (0.9 μSv [IQR, 0.1-12.2 μSv] vs 3.5 μSv [IQR, 1.3-6.3 μSv]; *P* = .14), or sonographers (0.0 μSv [IQR, 0.0-0.1 μSv] vs 0.2 μSv [IQR, 0.0-1.6 μSv]; *P* = .10) (Table 3).

**Table 3.** Personal Dose Equivalent per Case Received by Interventional Echocardiographers, Interventional Cardiologists, and Sonographers During Percutaneous Left Atrial Appendage Closures and Percutaneous Mitral Valve Repairs

Personal dose equivalent	Interventional echocardiographer	Interventional cardiologist	Sonographer	*P* value^a^	*P* value^b^	*P* value^c^
**Percutaneous left atrial appendage closure (n = 30)**						
Personal dose equivalent, median (IQR), μSv	10.6 (5.8-24.1)	3.5 (1.3-6.3)	0.2 (0.0-1.6)	<.001	<.001	<.001
Personal dose equivalent >20 μSv, No. (%)	9 (30.0)	1 (3.3)	4 (13.3)	.006	.12	.35
**Percutaneous mitral valve repair (n = 30)**						
Personal dose equivalent, median (IQR), μSv	10.5 (3.1-20.5)	0.9 (0.1-12.2)	0.0 (0.0-0.1)	<.001	<.001	<.001
Personal dose equivalent >20 μSv, No. (%)	8 (26.7)	2 (6.7)	1 (3.3)	.04	.03	.99

^a^ *P* value compares interventional echocardiographer and interventional cardiologist.

^b^ *P* value compares interventional echocardiographer and sonographer.

^c^ *P* value compares interventional cardiologist and sonographer.

During LAAO, interventional echocardiographers received a median radiation dose per case of 10.6 μSv (IQR, 5.8-24.1 μSv), which was 3.0-fold higher than the median radiation dose received by interventional cardiologists (3.5 μSv; IQR, 1.3-6.3 μSv) during the same procedure (*P* < .001) (Figure 2). Interventional echocardiographers received a radiation dose greater than 20 μSv in 30.0% of LAAO cases (n = 9), which was 12.4 (95% CI, 1.5-105.7) times more frequent than for interventional cardiologists (1 case [3.3%]; *P* = .006).

During TEER, interventional echocardiographers received a median personal dose equivalent of 10.5 μSv (IQR, 3.1-20.5 μSv). This radiation dose was 11.7-fold higher than the median dose received by interventional cardiologists (0.9 μSv; IQR, 0.1-12.2 μSv; *P* < .001) (Figure 2). During TEER, interventional echocardiographers received personal dose equivalents greater than 20 μSv in 8 cases (26.7%), which was 5.1 (95% CI, 1.0-26.4) times more frequent than interventional cardiologists, who received more than 20 μSv of radiation in only 2 cases (6.7%) (*P* = .04).

### Sonographer Radiation Exposure

During LAAO, the median radiation dose received by sonographers was 0.2 μSv (IQR, 0.0-1.6 μSv), which was significantly less than the dose for interventional echocardiographers (10.6 μSv; IQR, 5.8-24.1 μSv; *P* < .001) and interventional cardiologists (3.5 μSv; IQR, 1.3-6.3 μSv; *P* < .001). During TEER, the median radiation dose received by sonographers was 0.0 μSv (IQR, 0.0-0.1 μSv), which was also less than the median dose for interventional echocardiographers (10.5 μSv; IQR, 3.1-20.5 μSv; *P* < .001) and interventional cardiologists (0.9 μSv; IQR, 0.1-12.2 μSv; *P* < .001).

## Discussion

The primary results of this cross-sectional study of occupational radiation exposure indicate that interventional echocardiographers received significantly greater head-level radiation doses than interventional cardiologists during 2 commonly performed structural heart cases. In addition, in more than 25% of cases, the interventional echocardiographer received a dose that exceeded 20 μSv, which is roughly 10-fold higher than the previously reported mean personal dose equivalent received by interventional cardiologists across a variety of different procedures.^13^ In contrast, doses that exceeded 20 μSv were observed in 5% or fewer cases among interventional cardiologists during LAAO and TEER. Taken collectively, the findings of this study may have important occupational health implications for the rapidly expanding field of interventional echocardiography and for the structural heart team.

Despite previous work^3,4^ demonstrating the occupational radiation exposure risk to interventional cardiologists performing percutaneous coronary and structural heart interventions, similar data for interventional echocardiographers are lacking. Radiation exposure for individuals performing TEE was initially drawn from anesthesiologists who concomitantly performed echocardiography during transcatheter aortic valve replacements.^15^ In one of the largest studies to date involving a dedicated interventional echocardiographer during structural heart cases, Crowhurst et al^16^ demonstrated relatively high radiation doses across a variety of structural heart procedures, including transcatheter aortic valve replacement (TAVR), LAAO, TEER, and atrial or ventricular septal defect repairs. Although differences in radiation exposure were noted based on the occupation, no statistically significant difference was found between interventional echocardiographers and interventional cardiologists in pooled procedural analyses.^16^ Other studies have suggested that interventional echocardiographers receive higher radiation doses than interventional cardiologists^17^ and that dedicated shielding techniques for interventional echocardiographers may lower the occupational radiation exposure.^16,18,19^

Our data build on the pioneering work of Crowhurst et al^16^ and others^17,18,19^ in several important ways, which may explain the higher radiation doses experienced by interventional echocardiographers in the current analyses. First, the current study focused on 2 of the most common interventional echocardiographic procedures performed in the US (LAAO and TEER),^1,2^ whereas the work of Crowhurst et al^16^ focused primarily on TAVR procedures. Procedural characteristics associated with TAVR vs non-TAVR procedures, including for femoral angiography and angulation of the radiation source (particularly fewer steep right anterior oblique angles), have previously been associated with different radiation doses and may account for some of the observed between-study differences.^15,16^ That the current study focuses on LAAO and TEER (and excluded TAVRs) is of particular importance given that many facilities have transitioned away from a dedicated intraprocedural interventional echocardiographer during TAVR procedures and toward transthoracic procedural imaging, thereby limiting the generalizability of previous studies.^15,20^

Second, differences in positioning and shielding techniques may explain some of the observed differences. In the current study, the interventional echocardiographer was located at the head of the bed and directly faced the patient (and the primary source of scatter radiation) (Figure 1). This position contrasted with the study by Crowhurst et al,^16^ in which interventional echocardiographers were offset by approximately 45° and stood with their backs to the patient throughout the procedure. Positioning techniques in which the interventional echocardiographer has their back to the patient are not standard practice in many structural heart laboratories. Facing one’s back to the patient and structural heart team may also result in workflow limitations in manipulation of the TEE probe as well as team member communication.

Third, interventional echocardiographers in our study experienced markedly reduced radiation doses than in the study by Crowhurst et al^16^—comparatively 6-fold lower doses during LAAO and 4-fold lower doses during TEER.^16^ The lower radiation doses in the current study may in part be attributable to the use of state-of-the art fluoroscopy equipment, including noise reduction technology, which has been shown in at least 1 randomized clinical trial^21^ to reduce the operator radiation dose by 50%. Given these important positioning and procedural differences, direct comparisons in radiation doses between our study and the study by Crowhurst et al^16^ are limited.

Taken collectively, the observations of the current study inform the interventional echocardiography and the structural heart communities of the substantially higher risk of head-level radiation exposure of interventional echocardiographers during structural intervention procedures. The occupational risks depend on several factors, including the annual volume of TEE-guided fluoroscopic procedures performed, the radiation doses received during each procedure, the quality of the fluoroscopy imaging technology used, and the radiation safety practices of the institution in which the procedures take place. Additional studies are needed to determine whether thicker shields or alternative shielding configurations (such as suspended lead suits) would result in lower radiation doses than those observed. Without such technology, interventional echocardiographers may be vulnerable to even higher doses of radiation than reported herein, especially in catheterization laboratories that use older fluoroscopy equipment. We hope these data can help guide future research into risk mitigation methods to reduce the long-term low levels of ionizing radiation exposure, which have been associated with a number of adverse health effects.^3,4,5,6^

### Occupational Dose Limits

The radiation doses observed among interventional echocardiographers in this study can also be placed into context with respect to contemporary radiation dose limits set by regulatory agencies. In the US, the Occupational Safety and Health Administration sets an annualized limit for whole-body radiation exposure of 5 rem (50 mSv).^22^ Outside the US, occupational radiation exposure limits are more stringent; the International Commission on Radiologic Protection sets an annualized limit for whole-body radiation exposure of 20 mSv.^13^ On the basis of the procedural doses reported herein, it is doubtful that an interventional echocardiographer who uses the shielding techniques and state-of-the art fluoroscopy system described in this study would perform enough TEE-guided fluoroscopic procedures annually to reach these occupational limits.

However, whether adherence to annualized limits of radiation exposure is the preferable strategy remains controversial. The US Nuclear Regulatory Commission has suggested making “every reasonable effort to maintain exposures to ionizing radiation as far below the dose limits as practical.” This level has been termed *as low as reasonably achievable*, or *ALARA*. One of the driving reasons for this recommendation is the stochastic effect of ionizing radiation, which dictates that the probability of certain adverse effects of radiation, including cancer, is proportional to the exposure dose. As a result, many argue that there is no safe threshold of occupational radiation exposure. To this end, recent consensus statements and articles from professional societies^23,24,25^ have highlighted the issue of radiation exposure of interventional echocardiographers and have advocated for appropriate protection.

When physicians are exposed to annualized occupational doses well under the suggested limits, emerging evidence suggests that single procedural doses of occupational radiation exposure may have adverse biological effects. Lending support to this concept, El-Sayed et al^26^ recently demonstrated biologically detectable evidence of a DNA damage response, evident in blood samples drawn from physicians after performing a single fluoroscopically guided endovascular abdominal aortic aneurysm repair. The acute DNA damage response was detectable after cases in which the mean radiation dose to a chest dosimeter was 11 μSv. In direct comparison, evidence of an acute DNA damage response was not found in surgeons performing open abdominal aortic aneurysm repair not requiring fluoroscopy, suggesting that the DNA damage may indeed be mediated by radiation exposure.

### Sonographer Radiation Exposure

We also investigated the radiation exposure of another critical member of the structural heart team—sonographers. During LAAO and TEER, sonographers received lower doses of radiation than both interventional echocardiographers and interventional cardiologists. Sonographers in this study were exposed to median personal dose equivalents of only 0.2 μSv during LAAO and 0.0 μSv during TEER, demonstrating that it is possible for sonographers to perform their duties during fluoroscopic procedures with minimal personal radiation exposure. Given that sonographers wore the same lead shielding as interventional echocardiographers and were exposed to identical emitted radiation doses as part of the procedure, their low radiation doses are likely attributable to the ability of the sonographer to maintain a greater distance from the radiation source. These data are reassuring that with proper planning and equipment, effective preventive measures are practical and feasible for sonographers.

### Limitations

This study is limited in its generalizability by the single-center design and small sample size. Confirmation of the current observations in a larger series of cases performed at multiple centers is required. Radiation doses observed among interventional echocardiographers in this study are dependent on multiple factors associated with the use of shielding, including use of a protective scatter radiation absorbing shield (RADPAD), which was not recorded during the study interval. As a result, the study is limited in that results may not be generalizable to centers that use different shielding techniques, positioning of the interventional echocardiographer, and, importantly, positioning of the sonographer. In this regard, additional research is needed to determine optimal approaches to radiation safety among interventional echocardiographers. In addition, the arms and hands of interventional echocardiographers may be hypothesized to be particularly prone to high radiation exposure doses while manipulating the TEE probe. Radiation exposure to these high-risk body parts was not measured because this was beyond the scope of the present study.

## Conclusions

In this cross-sectional study, interventional echocardiographers were observed to have median head-level radiation doses that were 3.0-fold greater during LAAO and 11.7-fold greater during TEER than for interventional physicians. These comparatively increased radiation doses reveal a previously underrecognized occupational radiation exposure risk, which has important ramifications for the rapidly expanding field of interventional echocardiography.

## Notes

Supplement. **eAppendix.** Radiation Shielding
