# Supplementary material for: Comparison of Radiation Exposure Among Interventional Echocardiographers, Interventional Cardiologists, and Sonographers During Percutaneous Structural Heart Interventions
Source: JAMA Netw Open. 2022 Jul 7;5(7):e2220597. doi: 10.1001/jamanetworkopen.2022.20597 (PMC9264035; doi:10.1001/jamanetworkopen.2022.20597)
Supplement: Supplement. — eAppendix. Radiation Shielding [file jamanetwopen-e2220597-s001.pdf]

Supplementary Online Content

McNamara DA, Chopra R, Decker JM, et al. Comparison of radiation exposure among interventional echocardiographers, interventional cardiologists, and sonographers during percutaneous structural heart interventions. *JAMA Netw Open*. 2022;5(7):e2220597. doi:10.1001/jamanetworkopen.2022.20597

eAppendix. Radiation Shielding

This supplementary material has been provided by the authors to give readers additional information about their work.

© 2022 McNamara DA et al. *JAMA Network Open.*

eAppendix. Radiation Shielding

The lower section of the portable accessory lead shield (Mobile Shield WD257, Mavig, Munich, Germany) has width of 78 cm and height 95 cm and is comprised of a non-transparent steel body, which is not height-adjustable and has a lead equivalency of 1.0 mm Pb. It is intended to protect the lower body of the health care provider from scatter radiation. The upper section of this shield (width 70 cm), which is height adjustable, consists of a transparent lead acrylic panel having a lead equivalency of 0.5 mm Pb. The upper section of the shield was raised to a height that allowed the interventional echocardiographer to extend their arms over the shield to manipulate the TEE probe throughout the case (**Figure 1**). The time the top half of the lead shield was down to facilitate probe manipulation was minimized and was at the discretion of the interventional echocardiographer.

© 2022 McNamara DA et al. *JAMA Network Open.*
